# Supplementary material for: Expression of Selected miRNAs in Normal and Cancer-Associated Fibroblasts and in BxPc3 and MIA PaCa-2 Cell Lines of Pancreatic Ductal Adenocarcinoma
Source: Int J Mol Sci. 2023 Feb 10;24(4):3617. doi: 10.3390/ijms24043617 (PMC9961675; doi:10.3390/ijms24043617)
Supplement: Supplementary file 1 [file ijms-24-03617-s001.zip › ijms-2141910-supplementary.pdf]

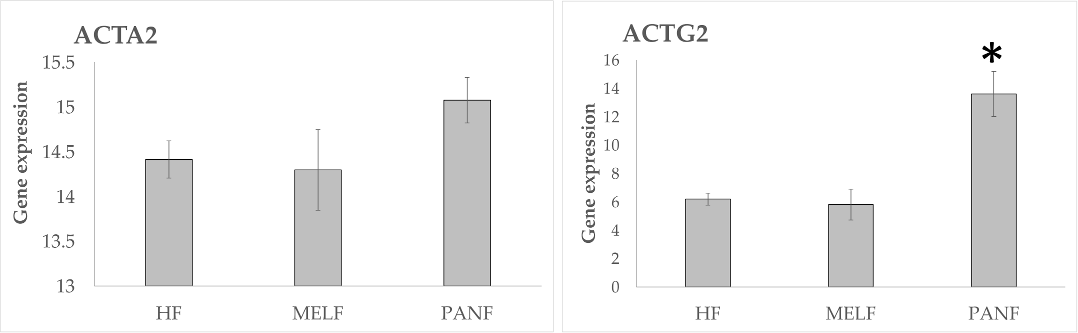

Supplementary Figure S1. While expression of *ACTA2* gene encoding α-smooth muscle actin was not significantly different in PANF in comparison to HF, the activity of *ACTG2* gene encoding intestinal smooth γ-muscle actin was significantly higher in PANF. Statistically significant differences between HF and other cell lines at adjusted p-value < 0.05 are marked by the asterisk.

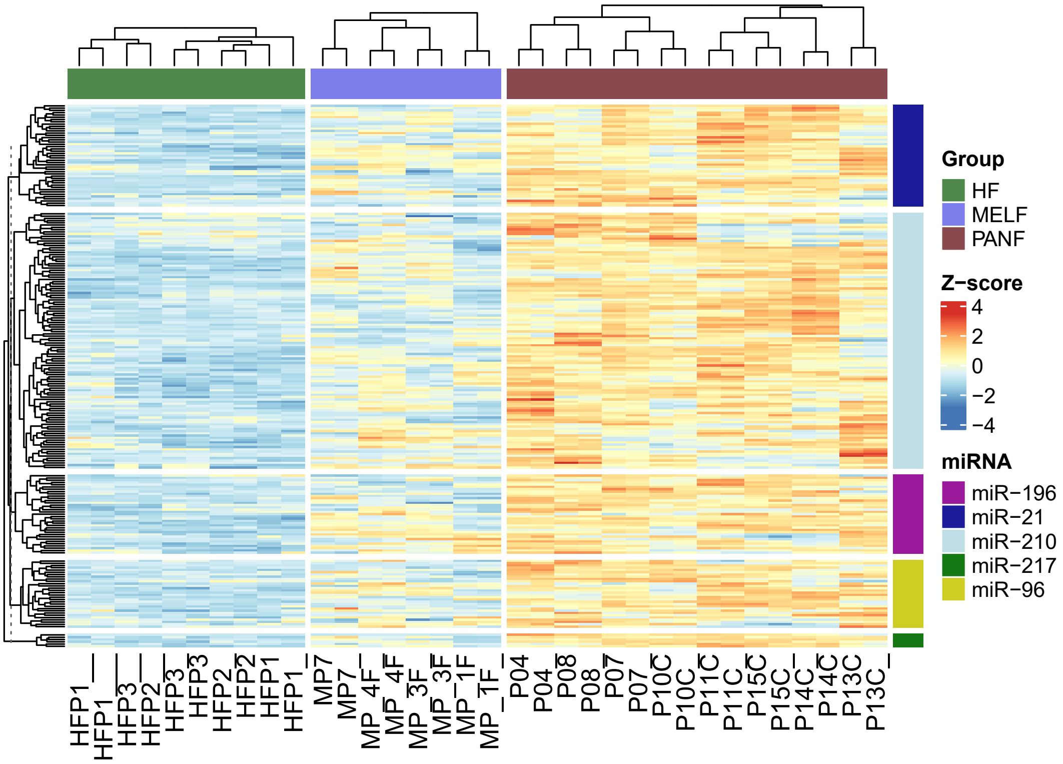

Supplementary Figure S2. Gene expression changes at the mRNA level for targets of miR-21, miR-210, miR-217, miR-96, and miR-196. For each miRNA, its experimentally validated targets were downloaded from the Tarbase v8 (http://microrna.gr/tarbase) database. The changes in gene expression of the targets that were also differentially expressed in the comparison of PANF and DF are displayed in the heatmap.

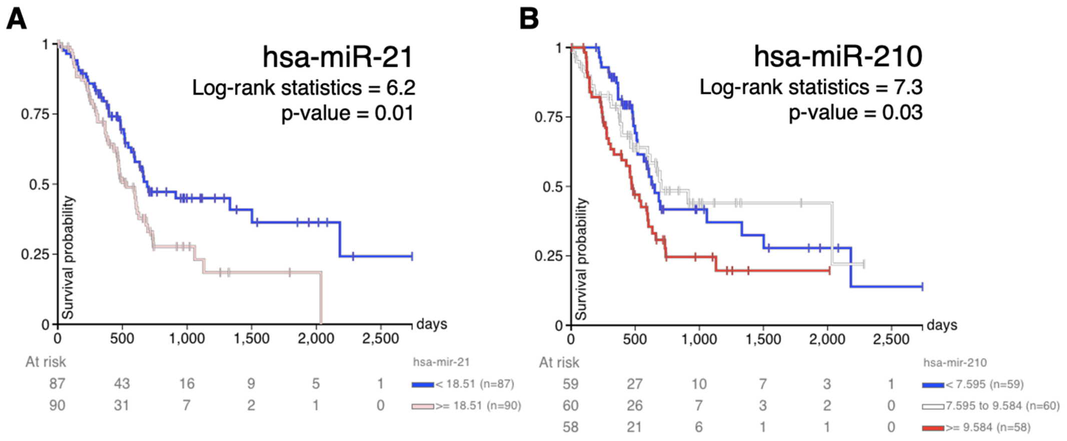

Supplementary Figure S3. Expression of miR-21 and miR-210 correlates with patient survival. Overall survival of the patients in the TCGA PAAD dataset depends on the expression of miR-21 and miR-210 in primary tumors. The data, Kaplan-Meier plots, and statistical tests were provided by the XENA portal (https://xenabrowser.net).

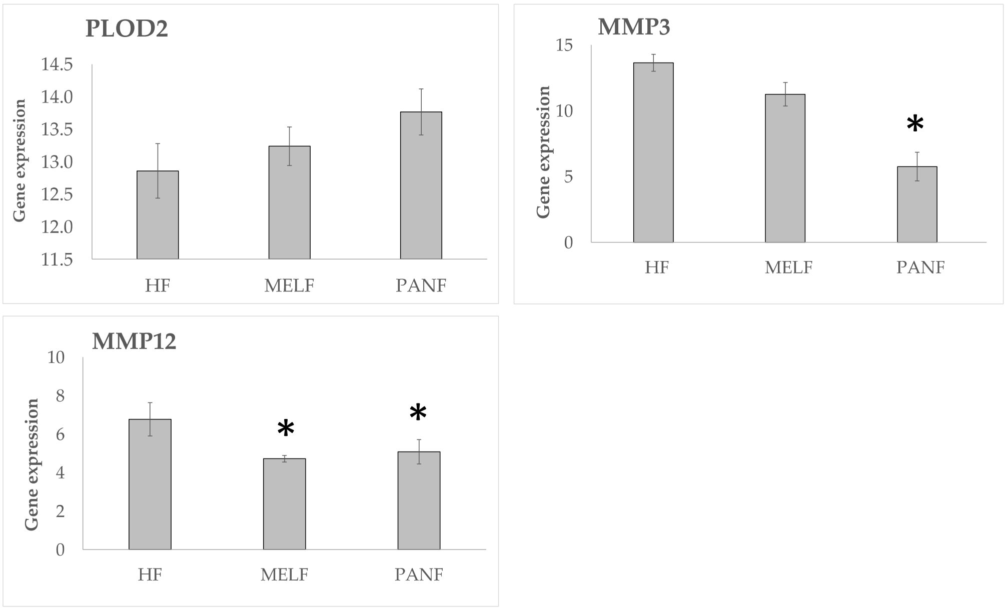

Supplementary Figure S4. Comparison of gene activity of PLOD2, MMP3, and MMP12 in normal fibroblasts and CAFs from the melanoma (MELF) and PDAC (PANF). Asterisks mark statistically significant differences between HF and PANF. Although the *PLOD2* gene is only insignificantly upregulated in PANF, it can affect crosslinking of collagen fibres and thus exert strong biological activity. Taken together with the downregulation of *MMP3/12* genes, which can reduce the remodelling of ECM, this data supports the desmoplastic character of PDAC stroma.

Supplementary Table S1. MicroRNAs log2 relative expression intensities in human cell lines compared to the tissue of the normal pancreas

Cell line	miRNAs fold change (Mean±SD)				
	**miR-21**	**miR-96**	**miR-196a**	**miR-210**	**miR-217**
HDF	2.41±0.56*<br>**FDR=0.004**	0.42±0.30<br>**FDR=0.004**	-0.63±0.18<br>**FDR=0.005**	1.36±0.24*<br>**FDR=0.03**	-0.60±0.26<br>**FDR=0.004**
HF.	3.99±0.27*<br>**FDR<0.001**	-0.84±0.40<br>**FDR<0.001**	-0.17±0.10<br>**FDR<0.001**	1.66±0.17*<br>**FDR<0.001**	-3.51±0.33*<br>**FDR<0.001**
PANF	3.32±0.18*<br>**FDR=0.03**	-1.11±0.22*<br>**FDR=0.01**	0.34±0.08<br>**FDR=0.03**	3.90±0.06*<br>**FDR=0.03**	-5.85±0.80*<br>**FDR=0.03**
BxPc3	2.13±0.38*<br>**FDR=0.03**	0.73±0.40<br>**FDR=0.03**	1.20±0.18*<br>**FDR=0.03**	1.69±0.24*<br>**FDR=0.03**	-4.85±0.80*<br>**FDR=0.03**
MIA PaCa-2	3.04±0.48*<br>**FDR=0.003**	1.27±0.37*<br>**FDR=0.003**	0.25±0.37<br>FDR=0.4	4.51±0.17*<br>**FDR=0.003**	-4.10±0.25*<br>**FDR=0.003**

* Minimally 2-fold higher or lower than in lysate from the normal pancreas at adjusted p-value FDR < 0.05.

Supplementary Table S2. Most significantly deregulated genes ($|log_2FC| > 1$, adjusted p-value < 0.05) for comparison PANF vs dermal fibroblasts

**Most significant genes**				
**Entrez ID**	**Symbol**	**Gene name**	**Log_2FC**	**Adjusted p-value**
8912	CACNA1H	calcium voltage-gated channel subunit alpha1 H	4.8	<0.0001
1004	CDH6	cadherin 6	4.18	<0.0001
9023	CH25H	cholesterol 25-hydroxylase	-5.86	<0.0001
160364	CLEC12A	C-type lectin domain family 12 member A	-3.53	<0.0001
22837	COBLL1	cordon-bleu WH2 repeat protein like 1	1.98	<0.0001
81035	COLEC12	collectin subfamily member 12	-6.59	<0.0001
1805	DPT	dermatopontin	-5.26	<0.0001
2018	EMX2	empty spiracles homeobox 2	-3.98	<0.0001
2019	EN1	engrailed homeobox 1	-2.66	<0.0001
80144	FRAS1	Fraser extracellular matrix complex subunit 1	-3.8	<0.0001
8322	FZD4	frizzled class receptor 4	2.68	<0.0001
3206	HOXA10	homeobox A10	-3.29	<0.0001
3223	HOXC6	homeobox C6	-3.3	<0.0001
3234	HOXD8	homeobox D8	-2.12	<0.0001
3490	IGFBP7	insulin like growth factor binding protein 7	2.88	<0.0001
10265	IRX5	iroquois homeobox 5	-4.42	<0.0001
3875	KRT18	keratin 18	6.68	<0.0001
284085	KRT18P55	keratin 18 pseudogene 55	6.59	<0.0001

54596	L1TD1	LINE1 type transposase domain containing 1	-1.74	<0.0001
4314	MMP3	matrix metallopeptidase 3	-6.92	<0.0001
4879	NPPB	natriuretic peptide B	4.72	<0.0001
5099	PCDH7	protocadherin 7	4.18	<0.0001
5308	PITX2	paired like homeodomain 2	-2.28	<0.0001
51316	PLAC8	placenta specific 8	-2.66	<0.0001
6275	S100A4	S100 calcium binding protein A4	-3.95	<0.0001
6335	SCN9A	sodium voltage-gated channel alpha subunit 9	5.24	<0.0001
23231	SEL1L3	SEL1L family member 3	3.31	<0.0001
58516	SINHCAF	SIN3-HDAC complex associated factor	1.85	<0.0001
5552	SRGN	serglycin	3.88	<0.0001
10617	STAMBP	STAM binding protein	1.12	<0.0001
6913	TBX15	T-box 15	-4.72	<0.0001
6943	TCF21	transcription factor 21	3.67	<0.0001
55504	TNFRSF19	TNF receptor superfamily member 19	-2.24	<0.0001
7291	TWIST1	twist family bHLH transcription factor 1	-2.84	<0.0001
79971	WLS	wntless Wnt ligand secretion mediator	3.75	<0.0001

Supplementary Table S3. The most significant GO terms for comparison PANF vs dermal fibroblasts, with added hypoxia and extracellular matrix organisation.

**50 most significant GO terms**						
**Rank**	**Accession**	**GO term**	**Category size**	**Overlap**	**Odds ratio**	**GSEA p-value**
1	GO:0048856	anatomical structure development	5541	500	2.327	<0.0001
2	GO:0007275	multicellular organism development	5073	470	2.339	<0.0001
3	GO:0032502	developmental process	5955	524	2.303	<0.0001
4	GO:0009653	anatomical structure morphogenesis	2467	285	2.635	<0.0001
5	GO:0048731	system development	4522	430	2.325	<0.0001
6	GO:0048513	animal organ development	3295	332	2.291	<0.0001
7	GO:0009888	tissue development	1892	226	2.61	<0.0001
8	GO:0009887	animal organ morphogenesis	980	147	3.245	<0.0001
9	GO:0072359	circulatory system development	979	146	3.219	<0.0001
10	GO:0032501	multicellular organismal process	7116	566	2.036	<0.0001
11	GO:0035295	tube development	571	96	3.579	<0.0001
12	GO:0016477	cell migration	1293	161	2.608	<0.0001
13	GO:0072358	cardiovascular system development	660	103	3.284	<0.0001
14	GO:0001944	vasculature development	653	102	3.285	<0.0001
15	GO:0040011	locomotion	1661	186	2.331	<0.0001
16	GO:0048870	cell motility	1430	167	2.421	<0.0001
17	GO:0051674	localisation of cell	1430	167	2.421	<0.0001
18	GO:0001568	blood vessel development	624	97	3.252	<0.0001
19	GO:0008283	cell proliferation	1993	210	2.199	<0.0001
20	GO:0048729	tissue morphogenesis	620	96	3.234	<0.0001
28	GO:0030198	extracellular matrix organisation	330	64	4.164	<0.0001
288	GO:0001666	response to hypoxia	336	41	2.34	<0.0001

Supplementary Table S4. Significantly deregulated genes (|log_2FC| > 1, adjusted p-value < 0.05) associated with hypoxia for comparison PANF vs dermal fibroblast

Genes associated with hypoxia				
**Entrez ID**	**Symbol**	**Gene description**	**Log_2FC**	**Adjusted p-value**
5244	ABCB4	ATP binding cassette subfamily B member 4	-1.04	0.005
94	ACVRL1	activin A receptor like type 1	-1.11	0.04
100	ADA	adenosine deaminase	-1.07	0.0002
133	ADM	Adrenomedullin	-2.25	<0.0001
51129	ANGPTL4	angiopoietin like 4	2.18	0.05
27063	ANKRD1	ankyrin repeat domain 1	2.9	0.0004
481	ATP1B1	ATPase Na+/K+ transporting subunit beta 1	-1.23	0.0003
650	BMP2	bone morphogenetic protein 2	1.27	0.003
664	BNIP3	BCL2 interacting protein 3	1.09	0.0002
847	CAT	catalase	-1.21	<0.0001
857	CAV1	caveolin 1	-1.32	0.001
100133941	CD24	CD24 molecule	-2.82	0.02
10370	CITED2	Cbp/p300 interacting transactivator with Glu/Asp rich carboxy-terminal domain 2	-1.07	0.009
6387	CXCL12	C-X-C motif chemokine ligand 12	-4.52	<0.0001
114757	CYGB	cytoglobin	2.81	0.001
1977	EIF4E	eukaryotic translation initiation factor 4E	1.19	0.0002
2026	ENO2	enolase 2	1.31	0.01
2034	EPAS1	endothelial PAS domain protein 1	-1.07	0.009
26355	FAM162A	family with sequence similarity 162 member A	1.38	0.007
56776	FMN2	formin 2	-1.43	0.0004
2627	GATA6	GATA binding protein 6	1.44	<0.0001
3091	HIF1A	hypoxia inducible factor 1 alpha subunit	0.69	0.09
29923	HILPDA	hypoxia inducible lipid droplet associated	0.8	0.01
3162	HMOX1	heme oxygenase 1	-1.7	0.006
3569	IL6	interleukin 6	2	0.09
3708	ITPR1	inositol 1,4,5-trisphosphate receptor type 1	2.04	<0.0001
3751	KCND2	potassium voltage-gated channel subfamily D member 2	1.2	0.02
3776	KCNK2	potassium two pore domain channel subfamily K member 2	-1.51	0.001
79625	NDNF	neuron derived neurotrophic factor	-4.29	<0.0001
4846	NOS3	nitric oxide synthase 3	1.11	0.03
50507	NOX4	NADPH oxidase 4	2.68	<0.0001
4879	NPPB	natriuretic peptide B	4.72	<0.0001
5228	PGF	placental growth factor	-1.42	0.03
5295	PIK3R1	phosphoinositide-3-kinase regulatory subunit 1	-1.07	0.004

5327	PLAT	plasminogen activator, tissue type	3.35	<0.0001
5328	PLAU	plasminogen activator, urokinase	2.65	0.003
5352	PLOD2	procollagen-lysine,2-oxoglutarate 5-dioxygenase 2	1.35	0.008
10631	POSTN	periostin	3.33	0.0005
5664	PSEN2	presenilin 2	1.36	0.0007
5743	PTGS2	prostaglandin-endoperoxide synthase 2	2.5	0.002
6095	RORA	RAR related orphan receptor A	1.19	0.06
6513	SLC2A1	solute carrier family 2 member 1	1.68	<0.0001
7010	TEK	TEK receptor tyrosine kinase	3.87	<0.0001
7042	TGFB2	transforming growth factor beta 2	2.72	<0.0001
7049	TGFBR3	transforming growth factor beta receptor 3	-2.89	<0.0001
7057	THBS1	thrombospondin 1	-1.21	0.0002
7291	TWIST1	twist family bHLH transcription factor 1	-2.84	<0.0001
7422	VEGFA	vascular endothelial growth factor A	2.25	<0.0001
126374	WTIP	WT1 interacting protein	-1.08	0.005

Supplementary Table S5. Significantly deregulated genes (|log_2FC| > 1, adjusted p-value < 0.05) associated with extracellular matrix for comparison PANF vs dermal fibroblast

Genes associated with extracellular matrix organisation				
**Entrez ID**	**Symbol**	**Gene description**	**Log_2FC**	**Adjusted p-value**
8728	ADAM19	ADAM metallopeptidase domain 19	2.51	<0.0001
11096	ADAMTS5	ADAM metallopeptidase with thrombospondin type 1 motif 5	-1.85	0.03
54507	ADAMTSL4	ADAMTS like 4	-1.19	0.07
84168	ANTXR1	anthrax toxin receptor 1	-1.24	0.0008
302	ANXA2	annexin A2	-1.02	0.02
146206	CARMIL2	capping protein regulator and myosin 1 linker 2	-1.16	0.003
1301	COL11A1	collagen type XI alpha 1 chain	5.34	<0.0001
80781	COL18A1	collagen type XVIII alpha 1 chain	-1.85	0.0007
1282	COL4A1	collagen type IV alpha 1 chain	2.69	<0.0001
1284	COL4A2	collagen type IV alpha 2 chain	2.81	<0.0001
1295	COL8A1	collagen type VIII alpha 1 chain	-1.45	0.07
1296	COL8A2	collagen type VIII alpha 2 chain	-1.28	0.01
1311	COMP	cartilage oligomeric matrix protein	-4.87	<0.0001
83716	CRISPLD2	cysteine rich secretory protein LCCL domain containing 2	-2.06	0.002
55790	CSGALNACT1	chondroitin sulfate N-acetylgalactosaminyltransferase 1	2.2	<0.0001
1513	CTSK	cathepsin K	-2.66	<0.0001
1514	CTSL	cathepsin L	-1.36	0.002
1634	DCN	decorin	-1.33	0.07
1805	DPT	dermatopontin	-5.26	<0.0001
133584	EGFLAM	EGF like, fibronectin type III and laminin G domains	1.09	0.03
2006	ELN	elastin	-2.93	0.005
2192	FBLN1	fibulin 1	-2.91	0.0002
2199	FBLN2	fibulin 2	-2.2	0.002
10516	FBLN5	fibulin 5	-1.15	0.005
23768	FLRT2	fibronectin leucine rich transmembrane protein 2	-2.14	0.0009
2296	FOXC1	forkhead box C1	-1.21	0.05
2303	FOXC2	forkhead box C2	2.54	<0.0001
2294	FOXF1	forkhead box F1	2.12	0.005
2295	FOXF2	forkhead box F2	-1.03	0.002
3038	HAS3	hyaluronan synthase 3	1.85	0.0004
5654	HTRA1	HtrA serine peptidase 1	-1.47	<0.0001
3672	ITGA1	integrin subunit alpha 1	1.17	0.01
3678	ITGA5	integrin subunit alpha 5	1.48	<0.0001
3689	ITGB2	integrin subunit beta 2	-1.41	0.02

284217	LAMA1	laminin subunit alpha 1	2.3	<0.0001
3910	LAMA4	laminin subunit alpha 4	-1.47	0.06
3915	LAMC1	laminin subunit gamma 1	1.01	0.0003
3918	LAMC2	laminin subunit gamma 2	2.52	0.0001
4016	LOXL1	lysyl oxidase like 1	-1.31	0.004
4060	LUM	lumican	-1.16	0.04
8076	MFAP5	microfibril associated protein 5	3.16	<0.0001
4320	MMP11	matrix metallopeptidase 11	1.12	0.02
4321	MMP12	matrix metallopeptidase 12	-2.85	<0.0001
4314	MMP3	matrix metallopeptidase 3	-6.92	<0.0001
4629	MYH11	myosin heavy chain 11	2.93	0.01
79625	NDNF	neuron derived neurotrophic factor	-4.29	<0.0001
4811	NID1	nidogen 1	-1.15	0.006
22795	NID2	nidogen 2	1.29	0.02
10609	P3H4	prolyl 3-hydroxylase family member 4 (non-enzymatic)	1.1	0.0006
5154	PDGFA	platelet derived growth factor subunit A	1.6	0.001
5156	PDGFRA	platelet derived growth factor receptor alpha	-1.06	0.0006
10631	POSTN	periostin	3.33	0.0005
8434	RECK	reversion inducing cysteine rich protein with kazal motifs	-1.12	0.005
222663	SCUBE3	signal peptide, CUB domain and EGF like domain containing 3	1.87	0.003
642658	SCX	scleraxis bHLH transcription factor	-2.11	0.007
6423	SFRP2	secreted frizzled related protein 2	-7.15	<0.0001
6662	SOX9	SRY-box 9	-1.4	0.08
10653	SPINT2	serine peptidase inhibitor, Kunitz type 2	3.37	<0.0001
6696	SPP1	secreted phosphoprotein 1	1.25	0.0001
7042	TGFB2	transforming growth factor beta 2	2.72	<0.0001
7057	THBS1	thrombospondin 1	-1.21	0.0002
79875	THSD4	thrombospondin type 1 domain containing 4	1.02	0.0001
3371	TNC	tenascin C	-2.49	0.0001
5212	VIT	vitrin	-3.41	<0.0001

Supplementary Table S6. Stem-loop primers for the miRNAs

miRNA name	Stem-loop Primer sequence
**miR-39 C. elegans**	GTCGTATCCAGTGCAGGGTCCGAGGTATTCGCACTGGATACGACTATTAC
**miR-21**	GTCGTATCCAGTGCAGGGTCCGAGGTATTCGCACTGGATACGACTCAACA
**miR-96**	GTCGTATCCAGTGCAGGGTCCGAGGTATTCGCACTGGATACGACAGCAAAAATGTG
**miR-196a**	GTCGTATCCAGTGCAGGGTCCGAGGTATTCGCACTGGATACGACCCCAACAACATG
**miR-210**	GTCGTATCCAGTGCAGGGTCCGAGGTATTCGCACTGGATACGACTCAGCCGCTGTC
**miR-217**	GTCGTATCCAGTGCAGGGTCCGAGGTATTCGCACTGGATACGACTCCAATCAGTTC

Supplementary Table S7. Real-time qPCR primers

Primer name	Primer sequence
Universal primer	ATCCAGTGCAGGGTCCGAGG
miR-39 C. elegans	GCGGCGGAGCTGATTTCGTCTTG
miR-21	GCGGCGGTAGCTTATCAGACTG
miR-96	GCGGCGGTTTGGCACTAGCAC
miR-196a	GCGGCGGTAGGTAGTTTCATGTTG
miR-210	GCGGCGGCTGTGCGTGTGACAG
miR-217	GCGGCGGTACTGCATCAGGAAC
